# Mechanobiology in cardiac physiology and diseases

**DOI:** 10.1111/jcmm.12027

**Published:** 2013-02-27

**Authors:** Ken Takahashi, Yoshihide Kakimoto, Kensaku Toda, Keiji Naruse

**Affiliations:** a Department of Cardiovascular Physiology, Graduate School of Medicine Dentistry and Pharmaceutical Sciences, Okayama UniversityOkayama, Japan; b Department of Medicine, Nagoya UniversityNagoya, Japan; c Department of Medicine, Okayama UniversityOkayama, Japan

**Keywords:** mechanoelectric feedback, mechanotransduction, mechanosensitive ion channels, arrhythmias, ischaemic/reperfusion injury, cardiogenesis, mechanomedicine

## Abstract

Mechanosensitivity is essential for heart function just as for all other cells and organs in the body, and it is involved in both normal physiology and diseases processes of the cardiovascular system. In this review, we have outlined the relationship between mechanosensitivity and heart physiology, including the Frank–Starling law of the heart and mechanoelectric feedback. We then focused on molecules involved in mechanotransduction, particularly mechanosensitive ion channels. We have also discussed the involvement of mechanosensitivity in heart diseases, such as arrhythmias, hypertrophy and ischaemic heart disease. Finally, mechanobiology in cardiogenesis is described with regard to regenerative medicine.

- Introduction
- Mechanobiology in normal cardiac physiology
  – Enhanced cardiac contraction in response to increased venous return
  – Mechanoelectric feedback (MEF)
  – Other physiological heart functions involved in mechanosensitivity
- Mechanotransduction
  – Mechanosensitive ion channels
- Mechanobiology in cardiovascular disease
  – Arrhythmias
  – Hypertension, hypertrophy and heart failure
  – Ischaemic/reperfusion injury and myocardial infarction
- Mechanobiology in cardiogenesis
- Conclusion

## Introduction

The heart is one of the organs whose function is intimately associated with mechanosensitivity. For instance, heart massage is performed as first aid for someone who has fallen down as a result of a cardiac arrest. Actually, this procedure is more than simply pushing out blood from inside the heart because it applies mechanical pressure stimulus to the heart, allowing it to regain normal rhythm. Although its success rate is not very high, the precordial thump, a rapid impact with a clenched fist to a specific place on the sternum, is a measure that may save peoples’ lives by reverting ventricular tachycardia into a normal sinus rhythm [1].

The phenomenon by which a mechanical stimulus to the heart affects its contraction is explained by the concept of mechanoelectric feedback (MEF) [2]. MEF is involved in heart rate regulation. Mechanosensitive ion channels are molecular devices for sensing mechanical stimuli, such as the atrial stretch in MEF. In this review, we discuss the mechanosensitive ion channels that are expressed in the heart.

Cardiovascular disease is the greatest cause of death worldwide, and it is likely to maintain this position until at least 2030 [3]. Mechanosensitivity is inseparably involved in normal cardiac physiology and with cardiovascular diseases that includes arrhythmias, hypertrophy and ischaemia–reperfusion injury. We discuss the current understanding of the mechanosensitivity of the heart and its relation with the pathophysiology of these diseases.

## Mechanobiology in normal cardiac physiology

In general, mechanotransduction is involved in cellular functions, such as proliferation, differentiation and apoptosis. Here, we discuss the organ-level response of the heart to mechanical stimuli.

### Enhanced cardiac contraction in response to increased venous return

When athletes are at a dead run during a 100 metre sprint or even when we run up the stairs, venous return to the heart increases. Fortunately, the vertebrate heart functions to pump the blood out by increasing cardiac contractile forces when large volumes of blood in the veins return back to the heart and this supports physical exercise. The Frank–Starling law of the heart, which has a history of over 100 years, states that ‘the volume of blood pumped by the heart each minute is determined almost entirely by the rate of venous return’. Although this basic principle has been studied intensely by numerous researchers (reviewed in [4, 5]), it is still being investigated.

This phenomenon is explained by the following three mechanisms: overlapping of actin and myosin filaments in the sarcomere, calcium sensitivity of myofilaments [6, 7] and titin-based passive tension [8]. Although these biophysically well-constructed theories elegantly explain the mechanisms underlying the Frank–Starling law, there is one other phenomenon that cannot be explained by these theories, *i.e*. the ‘slow force response’, which is a gradual increase in the heart contraction force in minutes, is seen after the immediate increase in contraction force in response to the stretching of cardiomyocytes [9–12]. Although its detailed mechanisms are still under discussion, the involvement of mechanosensitive ion channels expressed in the heart has been suggested [13–15].

### Mechanoelectric feedback (MEF)

Electrical excitation of myocytes is converted into mechanical movement of contraction by excitation–contraction coupling. This mechanical movement affects electrical excitation of myocytes. Periodic repeated contraction and relaxation of the heart is constantly modulated by this feedback system called mechanoelectric feedback (MEF). The concept of MEF is important because it is related to the development of arrhythmias and will be discussed later.

The fact that a stretch stimulus alters the membrane potential of cardiomyocytes was determined in the 1960s [16]. A stretch stimulus to the atrium prolongs an action potential's duration [15]. In general, when cardiomyocytes are stretched and/or pressure to the atrium or a ventricle is applied, the membrane potential of the cardiomyocytes depolarizes, the duration of an action potential shortens and the QT interval on an electrocardiogram shortens (see Lab's review in [2]). Stretch-activated channels are suggested to be involved in the MEF of the heart [17].

The orientation of cardiomyocytes differs because of the intramyocardial myocyte arrangement, and it is extremely difficult to examine the effects of a mechanical stimulus that is applied to cardiomyocytes during heart contraction. To deal with this problem, models that concurrently simulate the mechanical and electrical aspects of cardiac tissue have been developed using the finite element analysis technique [18]. Interestingly, the mechanism by which a precordial thump can revert arrhythmias into sinus rhythm has been estimated using a simulation model [19].

### Other physiological heart functions involved in mechanosensitivity

Atrial natriuretic peptide (ANP) is an important hormone that is involved in blood pressure regulation. ANP secretion is mediated by stretch-activated Cl channels [20]. K_ATP_ channels are also suggested to regulate ANP secretion [21]. It is intriguing that healthy women develop ventricular hypertrophy as a result of volume overload and increased stretch in the heart during pregnancy [22]. Stretch-activated c-Src kinase may be involved in this type of mechanically induced hypertrophy.

## Mechanotransduction

Each structure that forms the heart seems to be a device that senses mechanical stimuli, including the extracellular matrix, focal adhesion complexes, lipid bilayers, cellular orientation. In fact, each of these structures plays a role in mechanotransduction. In addition, numerous proteins are involved in mechanotransduction, including integrins, Rho kinase, PI3K, integrin-linked kinase, focal adhesion kinase, Src, extracellular signal-regulated kinase, MAP kinase, eNOS and others. These proteins are involved in cellular mechanotransduction pathways that mediate various heart responses, including arrhythmias, hypertrophy and ischaemic heart disease. Here, we have discussed the mechanosensitive ion channels that change their protein conformations in response to mechanical stimuli and induce successive responses of the cardiomyocytes.

### Mechanosensitive ion channels

**Fig. 1:**
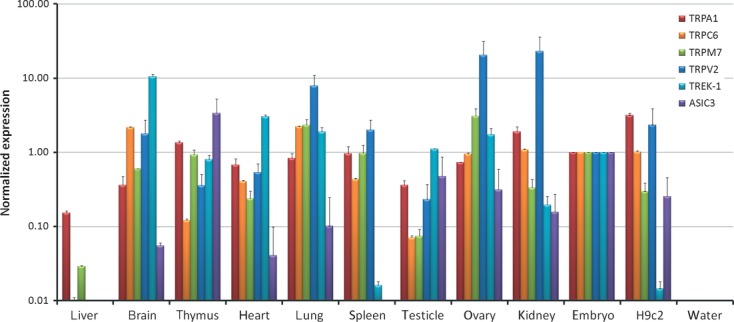
mRNA expressions of mechanosensitive ion channels determined by quantitative RT-PCR. Total RNA samples for liver, brain, thymus, heart, lung, spleen, testicle, ovary and kidney were obtained from 7–8 weeks old Sprague–Dawley rats. Total RNA sample for the embryo was obtained from midterm embryos of Sprague–Dawley rats. These RNA samples were purchased from Ambion. A total RNA sample for the rat ventricular cardiomyocyte cell line H9c2 was also obtained. TaqMan PCR primers were used for TRPA1, TRPC6, TRPM7, TRPV2, ASIC3 and TREK-1 channels' mRNAs. 18S ribosomal RNA was used as an internal control. Relative mRNA levels were calculated using ΔCt values (2∧(40 − ΔCt)) for each PCR run. Finally, the relative mRNA level was normalized to that of the embryo. All data are for three technical replicates.

Ion channels that are expressed in the heart and thought to be mechanosensitive are shown in Table 1. The expression levels of some of these channels determined by our group using quantitative RT-PCR are shown in Figure 1. As discussed below, TRPA1, TRPC6, TRPM7, TRPV2 and TREK-1 are involved in the heart's mechanosensitivity. Interestingly, the TREK-1 mRNA expression level is low in the cardiomyocyte cell line H9c2, which suggests that care should be taken when using H9c2 cells for cardiac research.

**Table 1:** Ion channels regarded as mechanosensitive in the heart

Channels	Species	Location
Sodium channels		
Na_v_1.5		
Na_v_1.6		
Potassium channels		
TREK-1	Rat	Cardiomyocyte
K_ATP_	Rat	Atrial myocyte
SAKCA	Chick	Ventricular myocyte
KCNQ	Rat	Cardiomyocyte
Calcium channel		
Ca_v_1.2	Human	Cardiomyocyte
Chloride channel		
CFTR	Rabbit	Atrial myocyte, SA node
ClC-3?	Rabbit	Atrial myocyte
Non-specific cation channels		
TRPA1		
TRPC1	Rat	Cardiomyocyte
TRPC6	Rat	Cardiomyocyte
TRPM4	Human	Purkinje fibre, SA node
TRPM7	Human	Atrial fibroblast
TRPP2		
TRPV2	Human	Cardiac muscle
TRPV4	Human	Atrial myocyte

**Fig. 2:**
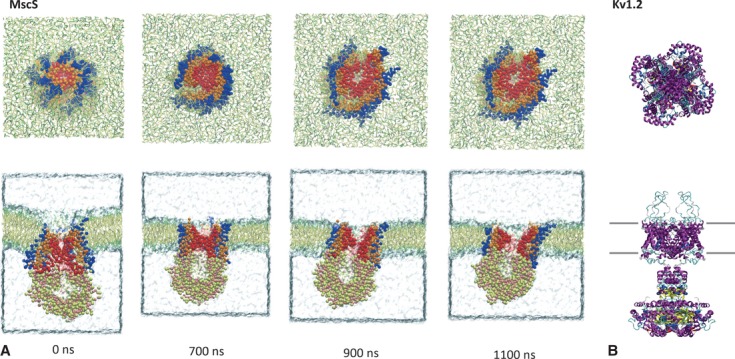
Structure of mechanosensitive ion channels. *Upper panels*: top views, *lower panels*: side views. (**A**) Opening of the bacterial mechanosensitive channel of small conductance (MscS, PDB accession number: 2OAU) in response to lipid bilayer stretch obtained by coarse-grained molecular dynamics simulation. MscS channel is embedded in lipid bilayers consisting of palmitoyloleoylphosphatidylcholine (POPC) and palmitoyloleoylphosphatidylethanolamine (POPE) lipids, which are shown in stick representation. The simulation box is filled with water molecules. Duration after applying a bilayer stretch is indicated in nanoseconds. Note that the channel's pore enlarges over time. (**B**) Three-dimensional crystal structure of mammalian Kv1.2 channel (PDB accession number: 3LUT) that putatively possesses mechanosensitivity.

Like other general ion channels, mechanosensitive ion channels change their conformations and become permeable to ions in response to multiple types of stimuli. Conformational changes in ion channels in response to mechanical stimuli have been well studied for the bacterial mechanosensitive channel MscL (mechanosensitive channel of large conductance). An example of mechanosensitive opening of MscS (mechanosensitive channel of small conductance) by coarse-grained molecular dynamics simulation carried out in our laboratory is shown in Figure 2A. As in this simulation, certain types of ion channels change their conformation in response to bilayer tension. Other types of channels sense mechanical stimuli by the interaction with cytoskeletal elements.

In vertebrates, several genes that encode for mechanosensitive ion channels have been identified. For TRPC3 channel, which is genetically close to TRPC1, its three-dimensional structure at 15 Å resolution was obtained by cryo-electron microscopy [23]. However, higher resolution structures, as with bacterial channels, have not yet been obtained, except for the Kv1.2 channel that is thought to have mechanosensitivity (Fig. 2B).

In patch clamp experiments, mechanosensitive ion channels in the heart were found to be stretch-activated channels that became permeable to cations in response to applying negative pressure to the cellular membrane [24]. Later, anion channels that responded to stretch stimulus and swelling [25] were discovered [26]. Cystic fibrosis transmembrane conductance regulator (CFTR), which is a mechanosensitive chloride channel [27], is expressed in cardiac myocytes [28]. Activation of myocardial CFTR channel upon reperfusion after cardiac ischaemia is involved in protection against myocardial injury induced by ischaemic reperfusion [29, 30]. Several potassium ion channels are known to be mechanosensitive. The TREK-1 channel is sensitive to mechanical [31, 32] and thermal [33] stimuli, in addition to arachidonic acid [32] and volatile anaesthetics [34]. Mechanosensitivity of K_ATP_ channels was reported in rat atrial myocytes [35]. K_ATP_ channels are involved in generating action potentials [36]. We found that one of the big potassium (BK) channels, SAKCA, expressed in chick cardiomyocytes was mechanosensitive [37–39]. The KCNQ channel responds to changes in cellular volume [40].

In addition to these potassium ion channels, TRP channels, which are non-selective cation channels, are also known to be mechanosensitive [41]. The relationships between TRP channels and heart diseases have been vigorously investigated [41–43]. TRP channels that are known to be mechanosensitive and are expressed in the heart, include TRPA1, TRPC1 [44–46], TRPC6 [45, 47], TRPM4 [48], TRPM7, TRPP2, TRPV2 [45, 49] and TRPV4 [50].

TRPA channels (alias, Painless) that are involved in pain sensing in *Drosophila* are known to be mechanosensitive. Interestingly, these channels are also expressed in the heart and are required for pressure sensing [51]. TRPC1 and TRPC6 channels are expressed in sinoatrial node cells [52]. Impaired touch and hearing sensations observed in TRPC3 and TRPC6 double-knockout mice are caused by abnormal mechanotransduction in sensory nerves and inner ear hair cells [53]. TRPC1 and TRPC6 are stretch-activated channels in the heart. TRPV4 channels are expressed in urothelial cell culture and are permeable to calcium ions in response to stretch stimuli [54]. TRPV4 channels in human corneal endothelial cells are permeable to calcium ions in response to hyposmotic stimulation [55]. TRPV4 channels in capillary endothelial cells have increased cellular calcium levels in response to stretch stimuli, which facilitates the reorientation of these cells [56]. TRPM4 channels are calcium-activated non-selective cation channels that are expressed in the sinoatrial node [57]. These channels are involved in transient inward currents (I_ti_) in the atrium [58]. TRPM7 channels are major calcium permeable channels in human atrial fibroblasts [59].

Gating of voltage-gated channels is also modulated by mechanical stimuli. Although their physiological role in the heart remains to be elucidated, the mechanosensitivity of Cav1.2 [60], Nav1.5 [61, 62] and Nav1.6 [63] has been reported in expression studies.

## Mechanobiology in cardiovascular disease

### Arrhythmias

Arrhythmias are heart diseases, in which the involvement of mechanosensitivity has been extensively studied. MEF theory indicates that interruption in the normal MEF cycle will lead to arrhythmias. Stretching of the atrium produces changes in action potential shapes and causes arrhythmia [64]. Mechanosensitive ion channels are thought to be directly involved in the process, in which cardiac tissue stretching induces changes in membrane potentials. TRPV4 channels might be involved in the development of arrhythmia *via* delayed after polarization [50]. TRPM4 channels are highly expressed in the cellular membranes of Purkinje fibres, and their overexpression has been suggested to cause progressive familial heart block type I [65]. A TRPM4 mutation causes conduction block in the heart [66]. It has been reported that an arrhythmia that developed as a result of hypoxia/reperfusion could be suppressed by the TRPM4 channel inhibitor 9-phenanthrol [67]. TRPM7 channels have been suggested to be involved in heart fibrogenesis during atrial fibrillation [59]. As mentioned above, several mechanosensitive ion channels have been suggested to be involved in the pathophysiology of arrhythmias. However, the development of effective cures needs additional research.

### Hypertension, hypertrophy and heart failure

It is known that TRPM4 expression is increased in hypertensive rats [58]. TRPM4 channels could be the cause of delayed after depolarization seen in these rats. In addition, TRPM4-deficient mice exhibit hypertension *via* increased catecholamine secretion [68]. Hypertension and valvular disease cause mechanical stimulation of cardiomyocytes, which induces hypertrophy of these cells *via* signal transduction pathways.

Hypertrophic responses are mediated by intracellular calcium levels. Store-operated channels (SOC) are regarded as the calcium source. TRPC1 and TRPC6 channels are candidate SOCs. Recently, the relationship between TRPC channels and cardiac hypertrophy has been revealed [42, 69]. TRPC channels are necessary mediators of pathological cardiac hypertrophy [70]. TRPC channels' expression is up-regulated during pressure overload to the heart [71]. In addition, TRPC6 channels are key components of a calcium-dependent regulatory loop involved in cardiac hypertrophy [72]. TRPC6 channels mediate hypertrophic responses in cardiomyocytes; however, they suppress fibrotic responses in cardiac fibroblasts [73]. Progressive pathological hypertrophy develops into heart failure. Stretch-induced apoptosis can lead to heart failure [74]. TRPC6 channel expression is up-regulated in failing hearts [75]. The mechanosensitivity of these channels, which may be involved in the pathophysiology of heart failure, should be the focus of a future study.

### Ischaemic/reperfusion injury and myocardial infarction

Ischaemic heart disease is a leading cause of death worldwide [3]. Short duration of ischaemia prior to sustained ischaemia can reduce injury caused by ischaemia–reperfusion injury [76]. This phenomenon is called ‘ischemic preconditioning’. Interestingly, stretch stimuli to the heart were found to have a preconditioning effect on ischaemia–reperfusion injury [77]. This ‘stretch preconditioning’ disappears when K_ATP_ channels are blocked [78, 79]. As mentioned earlier, K_ATP_ channels are mechanosensitive. On the other hand, CFTR channels, which are involved in cell volume regulation after osmotic swelling, play a role in ischaemic preconditioning [29, 80] and post-conditioning [30]. However, the interplay between K_ATP_ and CFTR mechanisms still remains to be elucidated. Further studies on the mechanisms involved in stretch preconditioning may lead to the development of new treatments for ischaemic heart diseases.

Prolonged ischaemia and successive reperfusion induce myocardial infarction, which may accompany arrhythmias. Cardiac mechanosensitivity has been suggested to be the cause of arrhythmogenesis in myocardial infarction. For example, a simulation study demonstrated that premature ventricular beats originated from the ischaemic border where mechanical strain was discontinuous, which may contribute to spontaneous arrhythmias [81]. TRPC6 protein expression is increased in rat myocardial infarction [82]. Future research may reveal whether increased TRPC6 expression is involved in the facilitated mechanosensitivity of cardiomyocytes at the border zone in myocardial infarction.

## Mechanobiology in cardiogenesis

During development, cells, tissues and organs assume their characteristic shapes by sensing mechanical stimuli and responding to them. The heart is an organ that first starts functioning in vertebrate embryos. Appropriate elasticity is required for calcium excitation and contraction of the cardiomyocytes [83]. In fact, substrate stiffness influences the heart rate contraction forces, the cytoskeletal structure and intracellular calcium levels in cardiomyocytes [84, 85].

Another key factor involved in cardiogenesis is cyclic stretching of cardiomyocytes, which is caused by pulsatile changes in cardiac internal pressure. Ott *et al*. performed interesting experiments, in which cardiac cells were reseeded onto a decellularized heart matrix [86]. When pulsatile perfusion was applied, thick viable cardiac muscles were obtained, whereas thin, weak muscles were obtained in a non-perfusion environment. Cyclic mechanical stretching influences both the expression and localization of connexin 43 [87]. Cyclic stretching also induces orientation of cardiomyocytes that is transverse to the stretch axis [88]. Thus, mechanical forces can affect intercellular communications *via* gap junction channels in the heart. Changes in blood flow patterns can impair cardiac septation and valve formation (reviewed in [89]).

In recent years, numerous attempts have been made to generate cardiomyocytes from embryonic stem cells, induced pluripotent stem cells and cardiac stem cells to find the means to repair adult hearts after heart attacks or other injuries [90–92]. Considering that the heart is an organ that is constantly exposed to mechanical stimuli, applying mechanical stimuli may be a key for generating robust cardiomyocytes from stem cells.

## Conclusion

The normal differentiation of tissues and organs, including the heart, is facilitated by mechanical stimuli during development. Cardiac mechanosensitivity is indispensable for normal heart physiology, as seen in the Frank–Starling law and MEF. Heart diseases have a significant impact on human health. Although the relationship between heart mechanosensitivity and the pathophysiologies of arrhythmias, hypertrophy and ischaemic heart disease is being revealed, further research needs to be conducted to apply this knowledge in finding effective remedies. Applying mechanical stimuli to stem cells is anticipated to contribute to the successful cellular induction of cardiomyocytes.
